# A safety study of high concentration and high frequency intravitreal injection of conbercept in rabbits

**DOI:** 10.1038/s41598-017-00683-x

**Published:** 2017-04-04

**Authors:** Jiaming Wang, Chunyan Lei, Lifei Tao, Quan Wu, Xiao Ke, Yiguo Qiu, Bo Lei

**Affiliations:** 1 Department of Ophthalmology, the First Affiliated Hospital of Chongqing Medical University, Chongqing Key Laboratory of Ophthalmology, Chongqing Eye Institute, Chongqing, China; 2 Shenzhen Eye Hospital, Affiliated Shenzhen Eye Hospital of Jinan University, Shenzhen Key Laboratory of Ophthalmology, Shenzhen, China; 3 Chengdu Kanghong Biotechnology Co. Ltd, Chengdu, China; 4 People’s Hospital of Zhengzhou University, Henan Eye Institute, Henan Eye Hospital, Henan Provincial People’s Hospital, Zhengzhou, China

## Abstract

The novel anti-VEGF drug conbercept has been used in the treatment of several retinal neovascular diseases. Owning to the alteration of the structure, the newest drug is capable of combining more molecular targets and present higher affinity to the angiogenesis promoting factors. However, it is unknown whether it will cause any unwanted effects like other anti-VEGF agents. We studied the short-term safety of high concentration and high frequency intravitreal injection of conbercept in rabbits. Intraocular pressure, fundus-photography, ERGs were applied. Retinal morphology, the amount of apoptotic cells and protein levels of IL-6, IL-8 and TNF-α in the aqueous humor were determined. Retinal proteomics was detected using tandem mass tags (TMTs) quantitative mass spectrometry. The difference of IOP, ERGs, protein levels of inflammatory factors among rabbits received conbercept and PBS was not significant (*P* > 0.05). Fundus photographs and retinal morphology of animals in the conbercept-injected groups mimic those observed in the PBS-injected groups. No TUNEL-positive cell was seen in the retinal ganglion cell layer in the conbercept-injected groups. Proteomics did not show significant changes of inflammation or apoptosis associated proteins in the conbercept-injected eyes. We conclude that intravitreal injection of high concentration and high frequency conbercept is well tolerated at least in a short-term in rabbits.

## Introduction

In the past decade, intravitreal injections of anti-vascular endothelial growth factor (VEGF) agents have been successfully used in the treatment of several retinal neovascular diseases which were incurable not long ago. Ranibizumab and bevacizumab were the two most extensively used anti-VEGF drugs. Recently, aflibercept and conbercept, two new anti-VEGF agents have been developed. Owning to the alteration of the drug structure, the novel anti-VEGF drugs are capable of combining more molecular targets and present higher affinity to the angiogenesis promoting factors^1, 2^. Clinically, the new drugs make it possible to prolong the interval between multiple injections^3^, are effective in some patients non-responsive to ranibizumab and bevacizumab^3, 4^, and even work in some severe patients with increased dosage. With a similar structure and effect to aflibercept, the newest anti-VEGF drug conbercept is a recombinant fusion protein composed of the second Ig domain of VEGFR1 and the third and fourth Ig domain of VEGFR2 to the constant region (Fc) of human IgG1. It is designed as a receptor decoy with high affinity for all VEGF isoforms and PlGF^1, 5, 6^.

Although clinical trials have proved anti-VEGF drugs exhibit satisfactory safety in many neovascular retinal diseases, patients undergo intravitreal injections still suffer from some unwanted adverse events, even if the occurrence is low. Moreover, long-term repeated injections increase the risk of the small-probability ocular side effects^7, 8^. In addition to the adverse events that caused directly by the injection procedure, some side effects may be associated with the drugs themselves. These drug-related side effects include endophthalmitis^9^, increase of intraocular pressure^7^, retinal toxicity, and decrease of retinal function^7, 10^. It has been reported that bevacizumab may lead to an increase of intraocular and systemic concentrations of IL-6 and IL-8 in patients^11, 12^ and a loss of retinal ganglion cells (RGCs) in rats^13^. Significant reduction of electroretinogram (ERG) a-wave and b-wave amplitudes in isolated bovine retinas after application of aflibercept has been reported, suggesting the drug might also affect retinal function^14^.

The improved anti-VEGF effect of aflibercept and conbercept may be attributed to expandation of their targets and increase of affinity. In addition to blocking VEGF-A receptor as ranibizumab and bevacizumab do, both aflibercept and conbercept combine VEGFB and placental growth factor (PlGF), which promote neovascularization and permeability of the blood vessel. However, VEGF-B and PlGF are involved in normal physiological functions^15, 16^. There are increasing concerns with regard to whether blocking these targets may cause any unwanted side effects. In addition, PlGF exerts a protective effect on retinal neuronal cells^16^ but it is still unknown whether it is an indispensable protective factor in the retina. Thus, one can never overemphasize the importance of the safety issue of the new agents, especially the safety concerns may be exaggerated in a diseased retina^17^.

We studied whether high dosage and high frequency intraocular application of conbercept would cause unwanted ocular adverse effects. The affinity, pharmacokinetic and systemic tolerability of intravitreal injection of conbercept in rabbit has been studied^18^. By using the same model, we evaluated the safety of intraocular administration of conbercept *via* morphological, functional and biological assessments. We also studied the retinal proteomics in the conbercept treated eyes.

## Results

### Intraocular pressure

**Figure 1:**
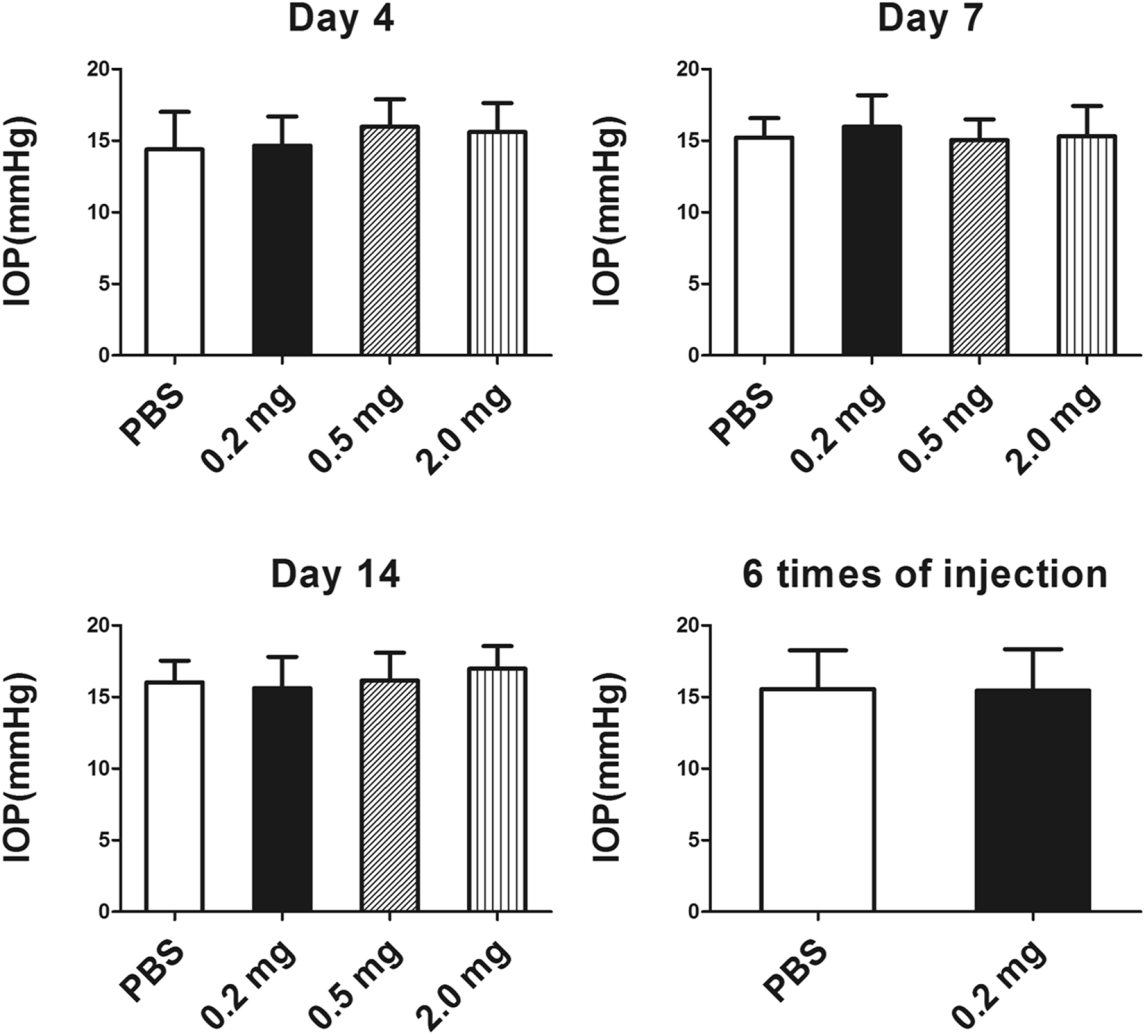
The intraocular pressure (IOP) in rabbits after a single injection of 50 μL PBS, 0.2, 0.5 or 2.0 mg of conbercept and 6 multiple injections of 50 μL of PBS or 0.2 mg of conbercept. There was no significance in IOP among the conbercept-injected groups and the PBS-injected groups (*p* > 0.05). The results are mean ± SD (n = 6).

The averaged IOPs (Fig. 1) after a single injection of PBS, or 0.2, 0.5, 2.0 mg of conbercept were 14.4 ± 1.3, 14.7 ± 2.2, 14.2 ± 1.9 and 15.1 ± 1.4 mmHg at day 4; 15.7 ± 1.8, 16.2 ± 2.4, 15,2 ± 1.3 and 15.8 ± 2.3 mmHg at day 7; 16.0 ± 2.0, 15.6 ± 1.8, 16.2 ± 1.9 and 16.9 ± 2.2 mmHg at day 14. The IOPs of the six-injection F and G groups were 15.3 ± 2.4 and 15.9 ± 1.9 mmHg on the 7^th^ day after the final weekly-injection. There was no significant difference (*p* > 0.05, n = 6) in IOP after the single injection of 0.2, 0.5, 2.0 mg and the six weekly injections of 0.2 mg conbercept compared to the PBS groups.

### Fundus examination

**Figure 2:**
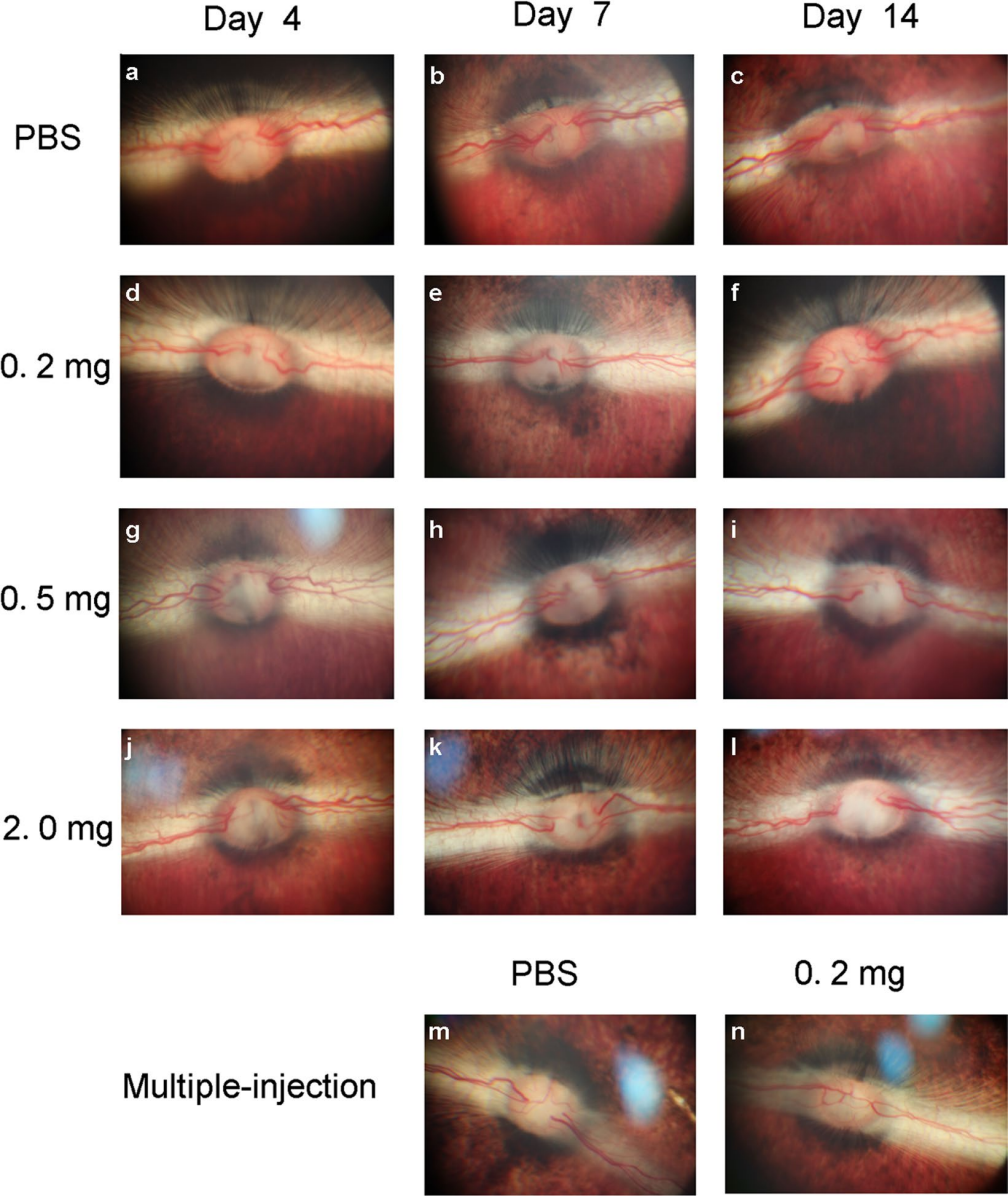
Fundus photographs taken on the 4^th^, 7^th^, 14^th^ day after a single injection of 50 μL PBS (**a–c**) or 0.2, 0.5, or 2.0 mg of conbercept (**d–l**) and on the 7^th^ day after the 6^th^ multiple injections of 50 μL PBS (**m**) or 0.2 mg of conbercept (**n**). The results observed in the conbercept-injected groups mimic those in the PBS-injected groups.

After a single intravitreal injection of different doses of conbercept, no signs of abnormalities or inflammation was seen in the fundus (Fig. 2d–l) when compared to the PBS-injected groups (Fig. 2a,b and c). The vitreous was clear and the vascular pattern appeared normal. No vascular narrowing, dilatation or tortuosity, retinal detachment, hemorrhage or optic nerve head changes were seen. Similar results were found in the six-weekly conbercept-injected group (Fig. 2m) and the PBS-injected group (Fig. 2n).

### ERG

**Figure 3:**
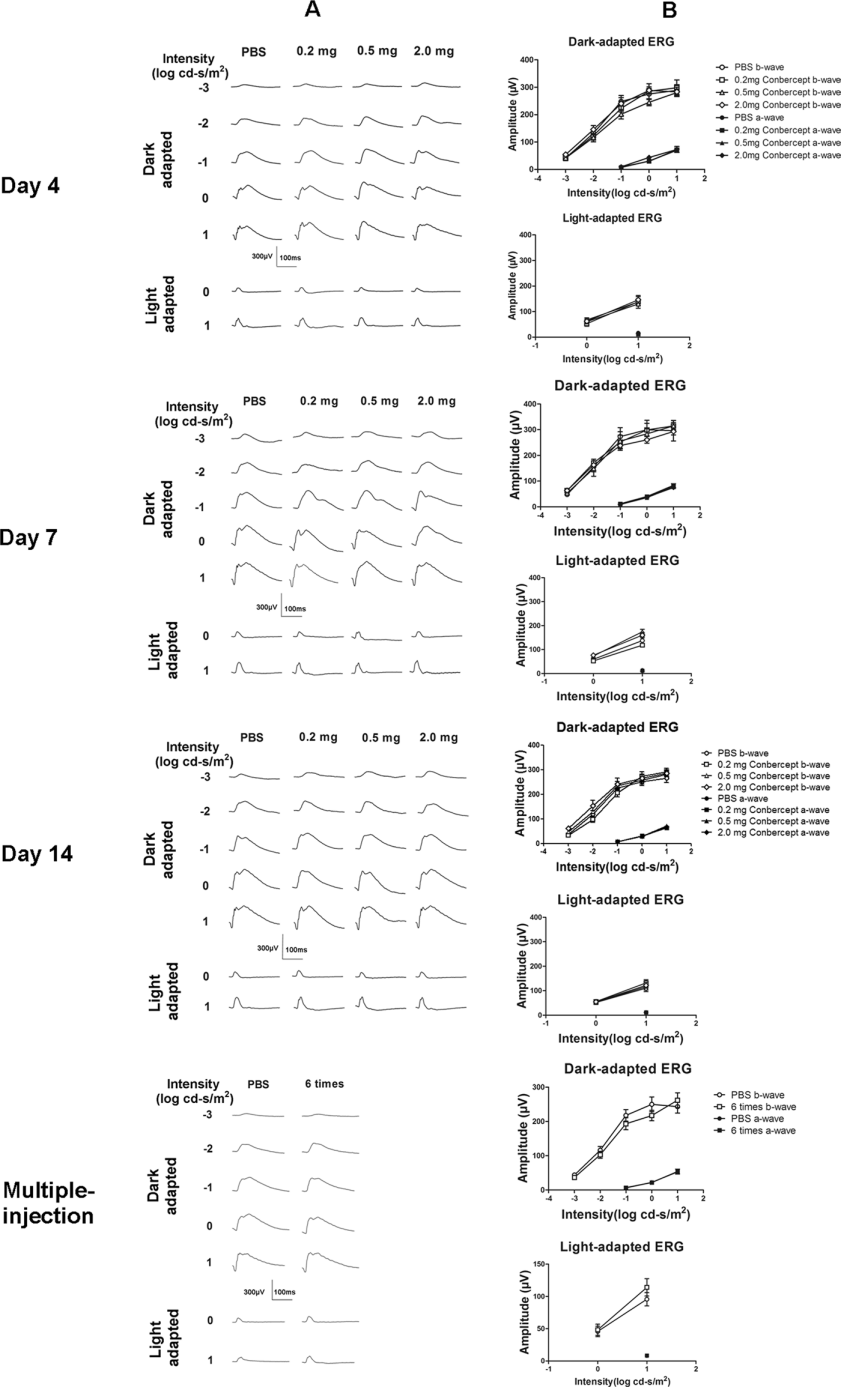
Dark- and light-adapted ERGs (**A**) and the amplitude *vs*. intensity profiles (**B**) obtained on the 4^th^, 7^th^, 14^th^ day after application of single intravitreal injections of 50 μL PBS or 0.2, 0.5, 2.0 mg of conbercept and on the 7^th^ day after the 6^th^ multiple injections of 50 μL PBS or 0.2 mg of conbercept. The amplitudes of a- and b-waves were not significantly different (*p* > 0.05). The results were mean ± SEM (n = 5).

The representative ERG responses (Fig. 3A) of the conbercept- and PBS-injected rabbits and the amplitude-intensity profiles (Fig. 3B) were exhibited. The ERG waveforms and the amplitudes of the averaged a- and b-wave recorded under various stimuli in the conbercept-injected groups mimicked those in the PBS-injected groups. There was no significant differences (*p* > 0.05, n = 5) between all the pairs in any of the recordings. The ERG showed no evidence of retinal functional damage after application of conbercept.

### Histology

**Figure 4:**
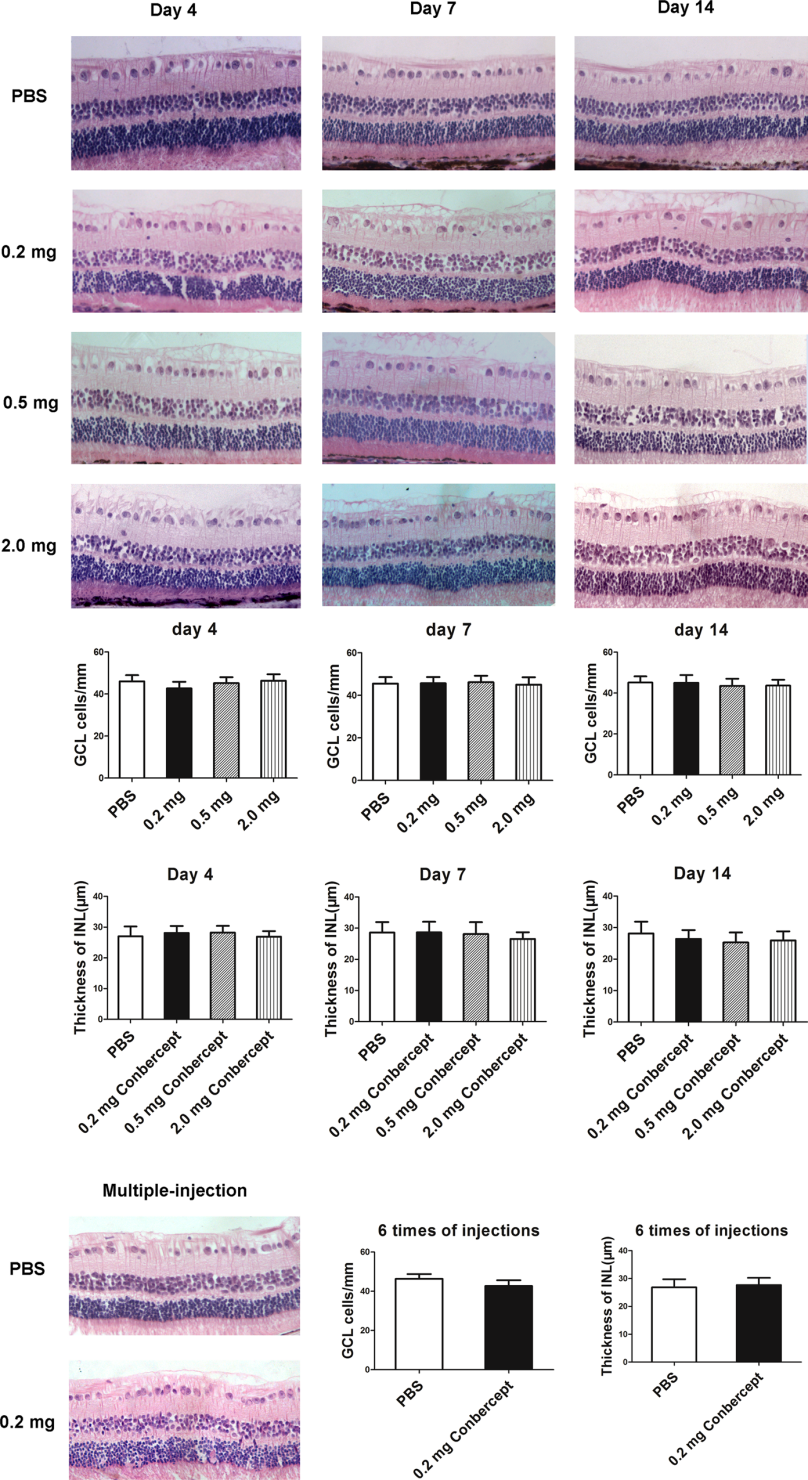
Retinal histology on the 4^th^, 7^th^, 14^th^ day after a single injection of 50 μL PBS or 0.2, 0.5, 2.0 mg of conbercept and on the 7^th^ day after the 6^th^ multiple injections of 50 μL PBS or 0.2 mg of conbercept. There was significant difference in the cell number in the ganglion ccell layer (GCL) and the thickness of inner nuclear layer (INL) (*p* > 0.05). The results were mean ± SEM (n = 3). Scale bar: 50 μm.

No apparent structural changes or sign of toxicity were observed in the conbercept-injected groups and the appearance of the retina were similar to the PBS control groups (Fig. 4). No sign of retinal degeneration, disorganization, thinning, cell loss, or hypocellularity was observed in these groups. The difference in the number of cells in the GCL in all the groups was not significant (*p* > 0.05, n = 3). There was no significant difference in the thickness of the inner nuclear layer (INL) among all the groups (*p* > 0.05, n = 3).

### TUNEL

**Figure 5:**
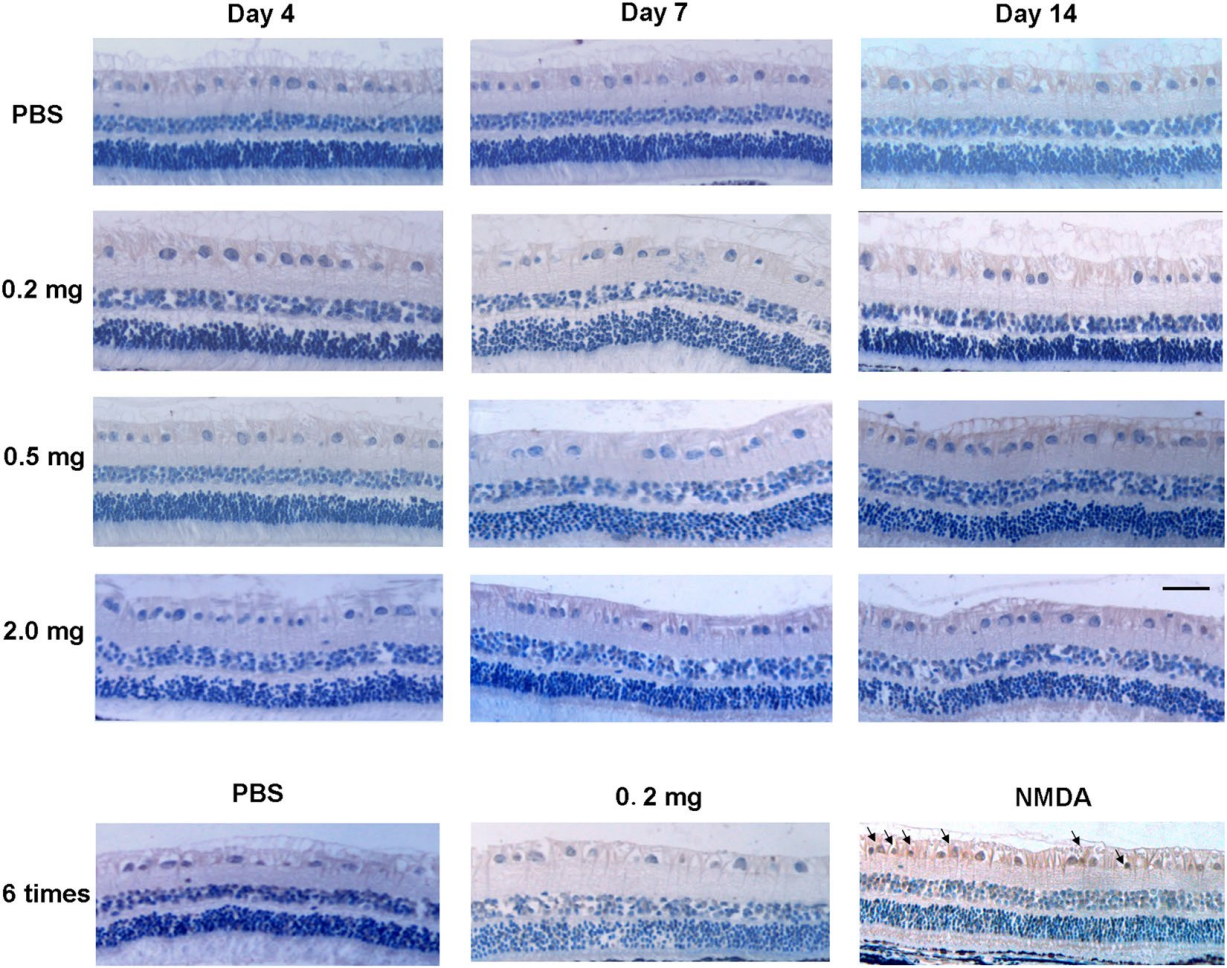
Detection of apoptotic cells in the ganglion cell layer (GCL) by TUNEL staining. Samples were collected on the 4^th^, 7^th^, 14^th^ days after a single injections of 50 μL PBS or 0.2, 0.5, 2.0 mg of conbercept and on the 7^th^ day after the 6^th^ multiple injections of 50 μL PBS or 0.2 mg of conbercept. No positive cells were observed in the GCL in the conbercept- and PBS-injected groups. TUNEL-positive cells were seen in the GCL and INL in the NMDA-injected eyes on the 7^th^ day after injection. Scale bar: 50 μm.

TUNEL-positive cells were not seen in the GCL of any animals injected with different doses and frequencies of conbercept and PBS (Fig. 5). No significant difference in apoptotic cell was observed among the conbercept injected and the PBS-injected groups. In the NMDA-injected group which was the positive control, positive cells were seen in the GCL.

### ELISA

**Figure 6:**
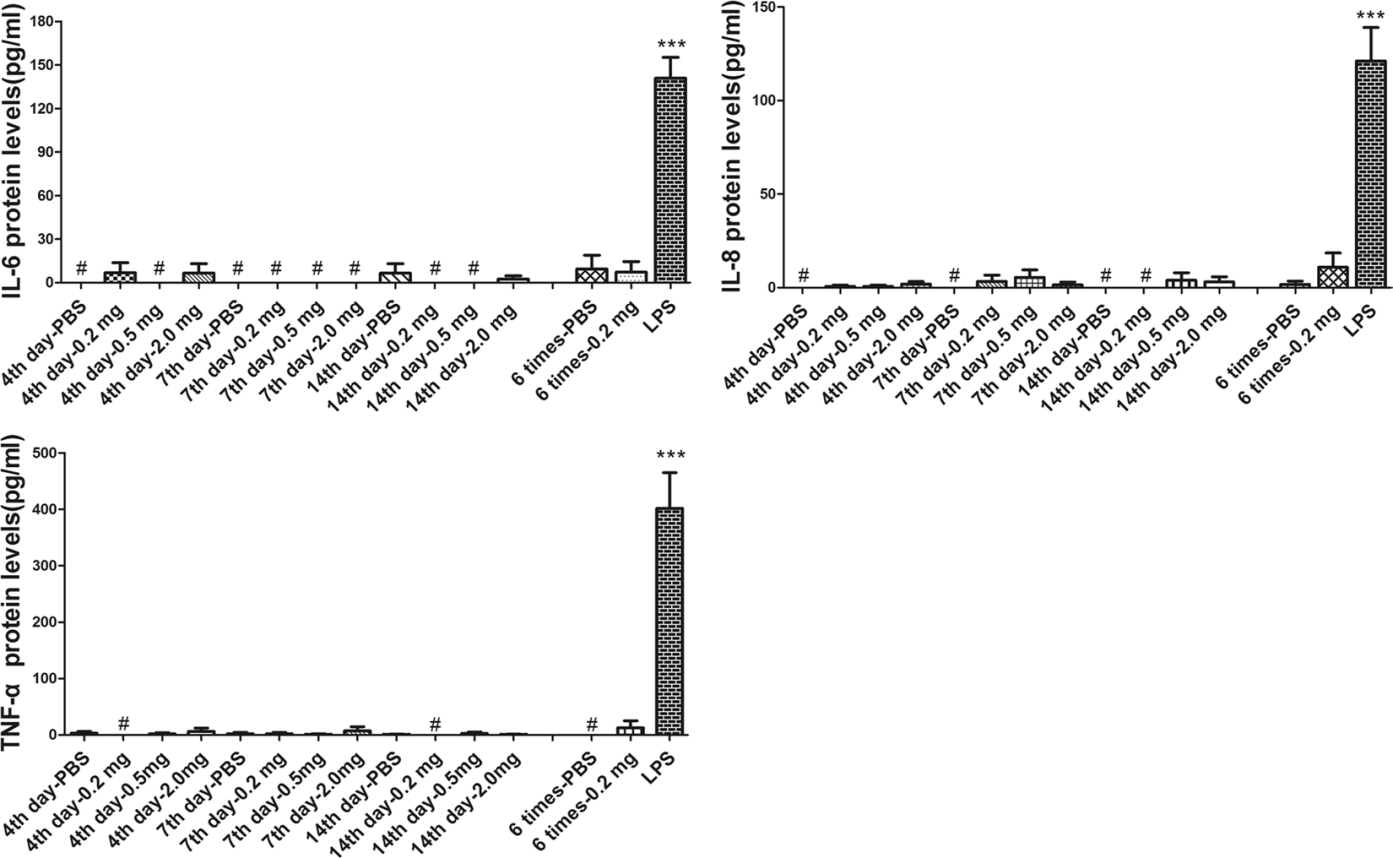
Protein levels of IL-6, IL-8 and TNF-α in the aqueous humor. Samples were collected on the 4^th^, 7^th^, 14^th^ day after single injection of 50 μL PBS or 0.2 mg, 0.5 mg, 2.0 mg of conbercept and on the 7^th^ day after the 6^th^ multiple injections of 50 μL PBS or 0.2 mg of conbercept as measured by ELISA. Positive control was collected 24 hours after injection of 100 μg of LPS. No statistically significant difference was found among the conbercept-injected groups and the PBS-injected groups. The protein levels of the three inflammation cytokines in the LPS-injected groups were significantly higher than all the other groups (*p* < 0.001). The results are mean ± SEM (n = 3~4). ^#^Undetectable.

Aqueous humor was collected at day 4, 7, 14 after a single intravitreal injection of different doses of conbercept or 50 μL of PBS and at day 7 after the 6^th^ weekly-injection of 50 μL PBS or 0.2 mg conbercept. The differences of the IL-6, IL-8 and TNF-α protein expression among the conbercept groups and the PBS groups was not significant (*p* > 0.05, n = 4) (Fig. 6). The concentrations of the cytokines in the LPS-injected group which served as a positive control were higher than those of the other groups (*p* < 0.001, n = 3~4).

### Proteomics analysis

Using the TMT quantitative mass spectrometry, a total of 6042 proteins were quantified from the rabbit retina on the 4^th^, 7^th^ and the 14^th^ day after intravitreal injection of 0.5 or 2.0 mg conbercept. Compared with the untreated control eyes, 250 proteins presented changes greater than 2 folds at either dosages or time point in the conbercept-injected eyes. Among them as shown in Tables 1, 2 and 3, 50 proteins were up-regulated, 57 were down-regulated and the rest 143 proteins showed inconsistent changes at different times or dosages.

**Table 1:** Significantly up-regulated proteins after intravitreal injection of conbercept.

Accession number	Gene symbol	Protein name	Fold change					
			Day 4–0.5 mg	Day 4–2.0 mg	Day 7–0.5 mg	Day 7–2.0 mg	Day 14–0.5 mg	Day 14–2.0 mg
655884547	FLT1	vascular endothelial growth factor receptor 1	3.85	3.32	2.57	18.35	3.46	6.73
655833765	MKRN2	probable E3 ubiquitin-protein ligase makorin-2 isoform X5	2.10	1.57	2.68	6.63	2.99	4.52
655855155	NFKBIZ, Mail	NF-kappa-B inhibitor zeta isoform X2	4.04	1.57	3.08	6.36	7.89	1.35
291405684	YPEL2	protein yippee-like 2	1.04	2.71	3.27	3.64	3.32	2.54
655826988		LOW QUALITY PROTEIN: 39 S ribosomal protein L20, mitochondrial-like	3.61	1.11	3.40	3.20	2.92	3.28
655841362	KLHDC3	kelch domain-containing protein 3	1.20	3.44	2.80	3.08	3.78	1.35
655879793	DLGAP3	LOW QUALITY PROTEIN: disks large-associated protein 3	1.14	1.63	2.57	3.05	1.83	1.54
655878481	RC3H2	roquin-2 isoform X4	1.97	1.95	1.56	2.99	1.58	1.86
655846130	BCAN	brevican core protein	3.23	3.07	1.97	2.80	2.96	2.45
655834754	RBM15B	putative RNA-binding protein 15B	1.60	1.68	2.87	2.80	3.38	3.77
291389567	ZFC3H1	zinc finger C3H1 domain-containing protein	2.41	2.29	1.50	2.69	1.76	1.31
655600655	VPS13A	vacuolar protein sorting-associated protein 13 A	1.20	1.57	1.88	2.56	1.79	1.35
315360675	EPT1, SELI	*ethanolaminephosphotransferase 1	1.20	1.57	1.89	2.53	1.96	1.35
655901171		maestro heat-like repeat-containing protein family member 1	2.26	1.68	1.89	2.52	1.21	1.65
655837568		RNA-binding protein 48-like	1.43	2.11	1.80	2.39	1.45	1.96
655866221	DNAJB12	LOW QUALITY PROTEIN: dnaJ homolog subfamily B member 12	1.62	2.12	2.29	2.34	1.40	1.49
129270090	CRYAA	*alpha-crystallin A chain	1.88	1.00	1.79	2.33	1.88	1.19
655787703	CMTM4	CKLF-like MARVEL transmembrane domain-containing protein 4	1.14	1.22	1.71	2.20	2.00	1.16
655834907	BAP1	ubiquitin carboxyl-terminal hydrolase BAP1	1.26	1.01	2.74	2.18	2.50	3.44
655882768	CRYBB1	beta-crystallin B1	1.93	1.28	2.14	2.12	2.02	1.55
655862947	GATM	LOW QUALITY PROTEIN: glycine amidinotransferase, mitochondrial	1.20	1.57	1.77	2.09	3.17	1.35
291416535	UVSSA	UV-stimulated scaffold protein A, partial	1.22	1.37	1.70	2.07	1.10	1.53
655883513		LOW QUALITY PROTEIN: carbonyl reductase [NADPH] 1-like	2.08	2.00	1.84	2.04	1.82	1.59
655896223	FAM118A	protein FAM118A	1.79	1.43	2.42	2.03	2.04	1.55
655850611		retinal-specific ATP-binding cassette transporter-like	1.73	1.95	2.20	2.00	2.02	1.88
655739906	BAZ2A	LOW QUALITY PROTEIN: bromodomain adjacent to zinc finger domain protein 2 A	2.10	1.49	1.64	1.96	1.67	1.89
126723630	LGALS3	*galectin-3	1.35	2.01	1.46	1.94	2.22	1.65
291387457	SRA1	steroid receptor RNA activator 1	1.10	1.46	1.32	1.93	1.81	2.39
655846332	GBA	glucosylceramidase isoform X2	2.26	1.65	1.66	1.86	1.65	1.36
655857145	BMPR1B	bone morphogenetic protein receptor type-1B isoform X2	2.35	2.57	4.93	1.85	5.11	2.57
655872717	ABCA5	ATP-binding cassette sub-family A member 5 isoform X2	2.16	1.55	2.17	1.83	2.27	2.05
655752526	CDK17	cyclin-dependent kinase 17	2.17	1.57	1.42	1.76	2.34	1.35
655771130	SAMD4B	protein Smaug homolog 2	1.20	1.57	2.13	1.76	1.95	1.35
655858544	PCDH10	protocadherin-10 isoform X2	1.50	2.18	1.72	1.67	1.59	1.23
655842878	CEP57L1	centrosomal protein CEP57L1 isoform X5	1.21	1.55	1.33	1.66	1.38	3.59
655605232	SORBS2	sorbin and SH3 domain-containing protein 2 isoform X9	1.61	2.24	2.23	1.60	1.12	1.84
655841271	TREML2	trem-like transcript 2 protein	9.55	1.57	2.51	1.59	2.81	1.35
291400631		ATPase inhibitor, mitochondrial-like	1.71	2.09	2.14	1.58	1.94	4.75
655727731	LETMD1	LETM1 domain-containing protein 1 isoform X2	2.53	2.27	1.79	1.57	1.83	1.35
291406313	MAPT	microtubule-associated protein tau isoform X11	5.27	1.50	3.98	1.48	2.58	4.30
291399405	MRTO4	mRNA turnover protein 4 homolog	1.36	1.20	2.30	1.45	1.22	1.59
655890274	OGG1	N-glycosylase/DNA lyase	1.86	1.57	1.92	1.38	1.88	2.89
291410889	PGER6	prostaglandin-E(2) 9-reductase	1.24	2.20	1.84	1.37	1.11	1.88
655835184	PXK	PX domain-containing protein kinase-like protein isoform X4	1.13	1.67	2.08	1.27	2.33	1.10
655601008	APOA1	apolipoprotein A-I isoform X1	1.11	2.85	1.09	1.24	1.34	1.31
655864723	ARPP19	cAMP-regulated phosphoprotein 19	1.14	1.40	1.22	1.20	1.22	2.06
655875742	PCSK1N	proSAAS	1.24	1.76	1.75	1.17	1.83	2.05
291405564	ASIC2	acid-sensing ion channel 2	1.59	1.68	2.17	1.15	1.14	1.02
147903853	ATP2A1, ATP2A3 SERCA1a	*sarcoplasmic/endoplasmic reticulum calcium ATPase 1	2.09	1.08	1.25	1.14	1.11	1.28
291398822	PARS2	probable proline–tRNA ligase, mitochondrial	1.75	1.58	2.15	1.11	1.40	1.06

*Means the proteins are not predicted proteins.

**Table 2:** Significantly down-regulated proteins after intravitreal injection of conbercept.

Accession number	Gene symbol	Protein name	Fold change					
			Day 4–0.5 mg	Day 4–2.0 mg	Day 7–0.5 mg	Day 7–2.0 mg	Day 14–0.5 mg	Day 14–2.0 mg
655828813	RAPH1	ras-associated and pleckstrin homology domains-containing protein 1	−0.77	−0.44	−0.88	−0.90	−0.74	−0.96
291416104	RGS8	regulator of G-protein signaling 8	−0.87	−0.83	−0.80	−0.89	−0.84	−0.39
291406868	PIK3IP1	phosphoinositide-3-kinase-interacting protein 1	−0.20	−0.40	−0.81	−0.89	−0.95	−0.23
655603509	LRRC4C LRRC4	leucine-rich repeat-containing protein 4 C	−0.93	−0.38	−0.98	−0.88	−0.97	−0.52
291384766	MPPED2	metallophosphoesterase MPPED2	−0.58	−0.25	−0.42	−0.88	−0.93	−0.46
291382897	TEX10	testis-expressed sequence 10 protein	−0.51	−0.67	−0.84	−0.88	−0.86	−0.27
655886298	ZNF629	zinc finger protein 629	−0.73	−0.49	−0.95	−0.84	−0.80	−0.94
291403838	MAP4K5	mitogen-activated protein kinase kinase kinase kinase 5	−0.70	−0.44	−0.90	−0.82	−0.89	−0.80
655876071	SPIN3	spindlin-3	−0.50	−0.15	−0.63	−0.82	−0.91	−0.98
655896069	MED15	mediator of RNA polymerase II transcription subunit 15 isoform X3	−0.36	−0.46	−0.54	−0.82	−0.71	−0.63
655605182	SNX25	sorting nexin-25	−0.12	−0.38	−0.63	−0.77	−0.83	−0.24
655633237	DMXL1	LOW QUALITY PROTEIN: dmX-like protein 1	−0.68	−0.42	−0.82	−0.77	−0.58	−0.68
291402173	SIPA1L2	signal-induced proliferation-associated 1-like protein 2	−0.98	−0.41	−0.88	−0.76	−0.99	−0.84
291408938	MED4	mediator of RNA polymerase II transcription subunit 4	−0.54	−0.40	−0.66	−0.74	−0.38	−0.55
655869905	UBE2G1	ubiquitin-conjugating enzyme E2 G1	−0.51	−0.36	−0.86	−0.74	−0.75	−0.54
655889686	DNAJC16	dnaJ homolog subfamily C member 16 isoform X2	−0.71	−0.67	−0.48	−0.71	−0.77	−0.51
655837812		Friend virus susceptibility protein 1-like	−0.61	−0.72	−0.48	−0.66	−0.49	−0.67
655899144	PGS1	LOW QUALITY PROTEIN: CDP-diacylglycerol–glycerol-3-phosphate 3-phosphatidyltransferase, mitochondrial	−0.44	−0.35	−0.73	−0.62	−0.61	−0.62
655842073	MB21D1	cyclic GMP-AMP synthase	−0.63	−0.57	−0.11	−0.61	−0.45	−0.70
655600047	DDX58	probable ATP-dependent RNA helicase DDX58 isoform X2	−0.47	−0.91	−0.43	−0.60	−0.66	−0.80
655901439		ras-related protein Rab-9A-like	−0.48	−0.63	−0.87	−0.59	−0.92	−0.96
655875606	CDK16	cyclin-dependent kinase 16 isoform X7	−0.34	−0.55	−0.83	−0.57	−0.22	−0.70
655847855	COL11A1	collagen alpha-1(XI) chain isoform X1	−0.60	−0.55	−0.50	−0.56	−0.82	−0.46
655844088	RMND1	required for meiotic nuclear division protein 1 homolog isoform X2	−0.32	−0.41	−0.71	−0.54	−0.64	−0.35
655902770		interferon-induced GTP-binding protein Mx1	−0.53	−0.96	−0.47	−0.53	−0.57	−0.85
655828284	STAT1	signal transducer and activator of transcription 1-alpha/beta	−0.51	−0.89	−0.50	−0.53	−0.60	−0.92
291384499	RRP8	ribosomal RNA-processing protein 8	−0.74	−0.53	−0.87	−0.51	−0.83	−0.38
655600642	RFK	riboflavin kinase	−0.46	−0.77	−0.56	−0.50	−0.49	−0.50
655871198	KRT15	keratin, type I cytoskeletal 15	−0.59	−0.92	−0.44	−0.49	−0.88	−0.88
655877487	ARMCX2	armadillo repeat-containing X-linked protein 2	−0.97	−0.68	−0.91	−0.49	−0.81	−0.34
655895404	DDX55	ATP-dependent RNA helicase DDX55 isoform X2	−0.81	−0.46	−0.61	−0.49	−0.76	−0.73
291409274		1,25-dihydroxyvitamin D(3) 24-hydroxylase, mitochondrial	−0.91	−0.74	−0.67	−0.44	−0.75	−0.64
291402773	ITGA11	integrin alpha-11	−0.57	−0.74	−0.38	−0.44	−0.67	−0.64
291389217	KRT1	keratin, type II cytoskeletal 1	−0.67	−0.78	−0.40	−0.44	−0.82	−0.78
298919207	RLA-A3	*MHC class I antigen-like precursor	−0.32	−0.55	−0.39	−0.40	−0.46	−0.77
655883687		cytosolic carboxypeptidase 1	−0.26	−0.43	−0.59	−0.40	−0.71	−0.30
284005498	AFF4, RA_m002_jsmFBA6Br	*AF4/FMR2 family member 4	−0.63	−0.36	−0.52	−0.40	−0.37	−0.41
291389201		keratin, type II cytoskeletal 6A-like	−0.45	−0.45	−0.38	−0.38	−0.70	−0.83
291394553	OSBPL3	oxysterol-binding protein-related protein 3	−0.44	−0.49	−0.23	−0.37	−0.41	−0.61
655812471		serine/arginine repetitive matrix protein 3-like	−0.42	−0.55	−0.51	−0.33	−0.62	−0.47
655730595	KRT72	keratin, type II cytoskeletal 72	−0.58	−0.71	−0.49	−0.33	−0.82	−0.74
655897183	CLASRP	CLK4-associating serine/arginine rich protein isoform X2	−0.48	−0.62	−0.51	−0.32	−0.38	−0.26
655603057	PRKRIR	52 kDa repressor of the inhibitor of the protein kinase	−0.93	−0.17	−0.80	−0.32	−0.73	−0.39
655840152	C12H6orf47	uncharacterized protein C6orf47 homolog	−0.31	−0.32	−0.28	−0.31	−0.20	−0.32
291389221	KRT3, CK-3, K3	keratin, type II cytoskeletal 3	−0.53	−0.74	−0.35	−0.31	−0.87	−0.55
291406083		keratin, type I cytoskeletal 14	−0.44	−0.39	−0.33	−0.30	−0.60	−0.70
655730654	KRT2	LOW QUALITY PROTEIN: keratin, type II cytoskeletal 2 epidermal	−0.60	−0.91	−0.32	−0.29	−0.80	−0.60
126722900		*lipophilin AL precursor	−0.29	−0.25	−0.41	−0.29	−0.26	−0.38
655891028		LOW QUALITY PROTEIN: D-dopachrome decarboxylase-like	−0.59	−0.24	−0.90	−0.27	−0.26	−0.38
291393010	LACC1	laccase domain-containing protein 1	−0.33	−0.38	−0.11	−0.26	−0.26	−0.36
126722998		*lipophilin CL2 precursor	−0.21	−0.26	−0.37	−0.25	−0.29	−0.50
291389193	KRT85	keratin, type II cuticular Hb5	−0.23	−0.22	−0.22	−0.22	−0.48	−0.22
291404267	SLC25A16	graves disease carrier protein	−0.25	−0.32	−0.66	−0.19	−0.54	−0.60
655872869	KRT34	LOW QUALITY PROTEIN: keratin, type I cuticular Ha4	−0.22	−0.20	−0.17	−0.18	−0.32	−0.19
655879141	UBL3	ubiquitin-like protein 3	−0.18	−0.26	−0.60	−0.15	−0.44	−0.63
291407186	MAP7D2	MAP7 domain-containing protein 2 isoform X7	−0.17	−0.48	−0.75	−0.13	−0.11	−0.49
291389187		keratin, type II cuticular Hb6	−0.21	−0.14	−0.18	−0.13	−0.27	−0.20

*Means the proteins are not predicted proteins.

^−^Means down-regulation.

**Table 3:** Significantly altered proteins with inconsistent changes after intravitreal injection of conbercept.

Accession number	Gene symbol	Protein name	Fold change					
			Day 4–0.5 mg	Day 4–2.0 mg	Day 7–0.5 mg	Day 7–2.0 mg	Day 14–0.5 mg	Day 14–2.0 mg
655874492	TPCN1	two pore calcium channel protein 1	1.68	2.40	1.16	2.88	−0.94	1.24
655846261	RIT1	GTP-binding protein Rit1	1.20	1.57	2.18	2.69	−0.81	1.35
655759055	RPS19BP1	active regulator of SIRT1	2.08	1.22	−0.90	2.66	−0.81	1.39
655868318	NSMCE4A	LOW QUALITY PROTEIN: non-structural maintenance of chromosomes element 4 homolog A	1.76	1.38	1.86	2.44	1.99	−0.67
655892261		transmembrane protein 120B	2.44	−0.91	1.92	2.33	1.69	1.32
291387997		epidermal retinol dehydrogenase 2	1.09	1.54	1.83	2.19	2.21	−0.93
655884071	HEATR3	HEAT repeat-containing protein 3	2.18	−0.92	1.31	2.14	1.68	−0.80
655870920	SP2	LOW QUALITY PROTEIN: transcription factor Sp2	−0.91	1.14	−0.66	2.11	−0.71	−0.67
291411045	PRR14	proline-rich protein 14	1.16	−0.50	2.38	2.10	−0.88	3.31
655880452	THAP1	THAP domain-containing protein 1 isoform X2	1.13	1.39	1.97	2.07	2.12	0.97
655854410	KALRN	kalirin isoform X20	4.29	1.57	1.44	1.97	−0.81	1.35
655828138	FKBP7	peptidyl-prolyl cis-trans isomerase FKBP7	−0.33	−0.65	1.55	1.87	−0.95	−0.98
291409542	INTS5	integrator complex subunit 5	−0.81	1.28	2.20	1.78	1.68	1.02
291410404	KCNJ13, KIR7.1	inward rectifier potassium channel 13 isoform X1	1.32	−0.81	2.01	1.72	1.63	1.74
655604456	STIM2	stromal interaction molecule 2 isoform X2	−0.29	−0.38	−0.86	1.71	1.35	−0.75
291409135	NR2F2	COUP transcription factor 2 isoform X3	1.20	2.43	−0.80	1.66	−0.81	1.35
655870532	MRPS23	28S ribosomal protein S23, mitochondrial isoform X2	1.20	3.51	1.64	1.64	−0.81	3.85
655886914	TRIM4	tripartite motif-containing protein 4 isoform X2	1.04	−0.77	1.35	1.61	−0.48	−0.70
291393060		heterogeneous nuclear ribonucleoprotein A1-like	−0.49	−0.44	−0.62	1.56	−0.80	1.13
291407324	LANCL3	lanC-like protein 3	−0.66	1.53	−0.72	1.53	−0.45	1.11
655605545	STON1	stonin-1 isoform X2	1.14	1.76	2.11	1.44	−0.92	1.16
655833182	MOBP	myelin-associated oligodendrocyte basic protein	1.30	1.49	−0.69	1.42	2.01	1.16
655603503	C1H11orf74	uncharacterized protein C11orf74 homolog	−0.49	1.53	1.34	1.41	1.37	1.29
655836760	ZNF532	zinc finger protein 532 isoform X3	2.50	1.12	2.21	1.38	−0.64	1.03
291403541	RBM23	probable RNA-binding protein 23	1.02	−0.44	−0.92	1.38	1.41	−0.63
655879659	OSCP1	protein OSCP1 isoform X4	1.29	−0.37	1.37	1.34	−0.61	1.22
291383827	TAGLN	transgelin	1.45	−0.47	−0.37	1.31	1.60	1.73
291401266	C15H4orf32	uncharacterized protein C4orf32 homolog	−0.48	1.85	1.16	1.24	1.65	−0.86
291394485	TIMM21	mitochondrial import inner membrane translocase subunit Tim21	−0.87	−0.39	−0.77	1.24	−0.33	−0.44
655899294	C3	complement C3 alpha chain isoform X1, partial	1.20	5.58	−0.44	1.22	1.55	1.15
284005533	MAP1A	*microtubule-associated protein 1A	1.28	1.96	2.14	1.21	−0.48	1.34
655601080	PRDM10	PR domain zinc finger protein 10 isoform X2	1.21	−0.48	1.31	1.20	−0.37	−0.68
655903015	ARHGEF1	LOW QUALITY PROTEIN: rho guanine nucleotide exchange factor 1	−0.52	−0.35	1.25	1.19	−0.94	−0.58
655859423	RGS7	regulator of G-protein signaling 7 isoform X4	−0.95	−0.48	−0.95	1.17	−0.87	1.11
655878265	ZBTB43	zinc finger and BTB domain-containing protein 43	1.03	−0.47	−0.91	1.16	2.52	−0.41
291383390	C1H9orf41	UPF0586 protein C9orf41 homolog	1.06	−0.54	−0.96	1.14	−0.98	−0.47
655832408	PIBF1	progesterone-induced-blocking factor 1	−0.32	−0.87	1.90	1.10	−0.67	−0.72
291401111	FGA	fibrinogen alpha chain	1.41	5.41	−0.58	1.09	1.15	1.01
291401007	TIAM1	T-lymphoma invasion and metastasis-inducing protein 1	−0.86	−0.45	−0.86	1.09	1.11	−0.95
291406107	NKIRAS2	NF-kappa-B inhibitor-interacting Ras-like protein 2	−0.47	−0.78	1.05	1.07	−0.71	1.29
291401109	FGB	fibrinogen beta chain	1.28	3.23	−0.84	1.07	−0.94	1.00
655601664	ALG9	alpha-1,2-mannosyltransferase ALG9 isoform X2	−0.76	−0.54	−0.93	1.06	−0.82	−0.13
291395292	PLCXD3	PI-PLC X domain-containing protein 3	−0.92	−0.45	−0.76	1.06	−0.90	−0.76
291402327	FBXO28	F-box only protein 28	1.06	1.24	−0.91	1.05	−0.71	−0.43
291402543	DSTYK	dual serine/threonine and tyrosine protein kinase isoform X2	−0.88	−0.50	1.21	1.04	−0.71	−0.57
655897677	KDELR1	ER lumen protein retaining receptor 1	1.28	−0.86	−0.49	1.04	−0.68	1.08
126723746	ALB	*serum albumin precursor	−0.91	2.52	−0.86	1.04	1.24	1.23
291413693	MRPL41	39S ribosomal protein L41, mitochondrial	−0.78	−0.33	1.28	1.04	1.13	1.32
655902607		phosphofurin acidic cluster sorting protein 2	−0.71	1.31	−0.73	1.03	−0.86	−0.38
655883487	PTPRE	LOW QUALITY PROTEIN: receptor-type tyrosine-protein phosphatase epsilon	−0.49	−0.64	−0.61	1.03	−0.69	1.52
655856160	FGG	fibrinogen gamma chain isoform X2	1.15	3.46	−0.72	1.02	−0.98	1.00
655862391	TRIP4	activating signal cointegrator 1 isoform X3	−0.54	−0.41	1.03	1.02	−0.90	−0.55
655603017		LOW QUALITY PROTEIN: transmembrane protease serine 13-like	1.20	1.29	−0.41	1.00	1.82	1.07
655608615	PSME4	proteasome activator complex subunit 4	1.63	2.28	1.19	−0.98	1.44	1.76
655602331	LIPT2	putative lipoyltransferase 2, mitochondrial	−0.79	−0.78	−0.40	−0.98	1.38	−0.68
655805767	RRN3	RNA polymerase I-specific transcription initiation factor RRN3 isoform X2	1.16	1.06	2.04	−0.97	−0.71	1.40
157787195	TPM2	*tropomyosin 2 (beta)	3.88	1.74	1.23	−0.97	1.18	1.22
655807516		LOW QUALITY PROTEIN: THUMP domain-containing protein 1-like	−0.97	−0.46	1.00	−0.96	−0.96	−0.59
291389041	PAK7	serine/threonine-protein kinase PAK 7	−0.74	−0.49	1.11	−0.96	−0.90	1.09
291406137	NAGLU	alpha-N-acetylglucosaminidase	1.59	−0.50	1.11	−0.95	−0.48	−0.70
655858943	USP6NL	LOW QUALITY PROTEIN: USP6 N-terminal-like protein	−0.88	−0.57	−0.98	−0.95	1.00	−0.46
655601434	FXYD6	FXYD domain-containing ion transport regulator 6 isoform X4	1.31	1.36	2.10	−0.95	1.53	1.34
126723638	PAPSS2	*bifunctional 3′-phosphoadenosine 5′-phosphosulfate synthase 2	−0.38	−0.76	1.50	−0.94	1.23	1.54
655897422		uncharacterized protein C16orf74 homolog	−0.50	−0.62	1.11	−0.94	−0.86	−0.48
655801810	RBFOX1	RNA binding protein fox-1 homolog 1	−0.62	−0.46	1.05	−0.94	−0.61	−0.83
655879231	RFC3	replication factor C subunit 3 isoform X2	−0.97	−0.42	1.01	−0.94	1.08	−0.63
655827806	LRP2	low-density lipoprotein receptor-related protein 2	2.13	1.57	−0.80	−0.94	−0.81	1.35
126723185	ADCY10, SAC	*adenylate cyclase type 10	1.20	3.24	1.29	−0.94	−0.81	1.35
291394610	NOD1	nucleotide-binding oligomerization domain-containing protein 1	1.20	1.57	2.70	−0.94	−0.81	1.35
655897113	ERCC1	DNA excision repair protein ERCC-1	1.20	1.57	2.08	−0.94	−0.81	2.86
291408033	TCEAL5	transcription elongation factor A protein-like 5	1.10	−0.19	1.07	−0.93	−0.76	1.58
291395159	FAM134B	protein FAM134B	−0.90	−0.37	1.20	−0.92	1.03	1.04
291403287	ACTC1	actin, alpha cardiac muscle 1	3.87	1.62	1.21	−0.92	1.24	1.59
291393596	ELP6	elongator complex protein 6 isoform X2	1.25	−0.45	1.00	−0.91	−0.93	1.04
291410032	CBR3	carbonyl reductase [NADPH] 3	−0.62	−0.45	−0.99	−0.91	−0.94	1.47
130493079	TNNI2, TnI	*troponin I, fast skeletal muscle	2.18	1.10	−0.90	−0.91	−0.82	1.05
655605874	KCNIP3	calsenilin isoform X3	1.04	−0.47	−0.87	−0.90	−0.96	1.25
291400645	QTRTD1	queuine tRNA-ribosyltransferase subunit QTRTD1	−0.56	1.68	−0.40	−0.90	−0.87	−0.55
291412904	#N/A	mitogen-activated protein kinase 8 isoform X1	1.28	−0.33	1.14	−0.89	1.27	1.37
655895776	SYVN1	LOW QUALITY PROTEIN: E3 ubiquitin-protein ligase synoviolin	1.38	−0.31	−0.98	−0.87	−0.93	−0.55
655663205	ZNF346	zinc finger protein 346 isoform X2	1.96	1.87	2.23	−0.87	1.30	1.92
655866440	PBLD	phenazine biosynthesis-like domain-containing protein isoform X2	−0.80	2.20	2.75	−0.87	2.38	6.72
156119398	MYL1	*myosin light chain 1/3, skeletal muscle isoform	2.84	−0.76	−0.94	−0.85	−0.92	−0.99
291403770	GEMIN2	gem-associated protein 2 isoform X1	1.25	−0.49	1.13	−0.85	−0.80	1.11
291404856	TRUB1	probable tRNA pseudouridine synthase 1	1.21	1.71	2.13	−0.84	−0.77	1.36
655853659	HRG	histidine-rich glycoprotein	−0.90	2.46	−0.73	−0.84	1.25	1.19
655602610	APBB1	amyloid beta A4 precursor protein-binding family B member 1 isoform X6	−0.88	−0.45	−0.59	−0.84	1.99	−0.87
655861319	GLRX2	glutaredoxin 2 isoform X2	−0.34	−0.54	−0.70	−0.83	−0.61	1.42
291392203	SMARCAL1	SWI/SNF-related matrix-associated actin-dependent regulator of chromatin subfamily A-like protein 1	1.85	−0.56	2.02	−0.83	2.36	1.47
655868632		myosin-2	2.36	−0.85	−0.91	−0.82	−0.88	−0.87
291404152	FUT11	alpha-(1,3)-fucosyltransferase 11	1.03	1.05	−0.91	−0.81	−0.80	−0.39
291416252	EXOSC4	exosome complex component RRP41	−0.83	−0.42	−0.84	−0.81	−0.80	1.11
291402469	HSD11B1	corticosteroid 11-beta-dehydrogenase isozyme 1	−0.48	−0.34	−0.38	−0.81	1.08	−0.57
655838173	GPR98	G-protein coupled receptor 98	1.19	−0.47	1.52	−0.80	1.17	1.44
655599632	STX17	syntaxin-17	−0.28	1.18	−0.96	−0.79	−0.70	−0.95
655848676	FUBP1	far upstream element-binding protein 1 isoform X14	−0.82	−0.45	−0.80	−0.78	−0.97	1.00
655874201	ANAPC5	anaphase-promoting complex subunit 5 isoform X3	−0.71	−0.47	1.54	−0.76	−0.76	1.45
655841323	GLTSCR1L	GLTSCR1-like protein isoform X2	−0.99	−0.48	−0.71	−0.75	1.07	−0.84
655889301	PPL	periplakin	−0.85	−0.45	1.78	−0.73	−0.82	1.33
291406958	PPTC7	protein phosphatase PTC7 homolog	−0.46	−0.41	1.04	−0.73	−0.87	−0.87
655758942	MYBPC1	myosin-binding protein C, slow-type, partial	1.43	1.39	−0.85	−0.72	−0.91	2.17
291393095	COMMD6	COMM domain-containing protein 6 isoform X2	−0.42	−0.64	1.00	−0.71	1.07	−0.63
291402968	PIGB	GPI mannosyltransferase 3	−0.90	−0.86	−0.63	−0.70	1.00	−0.34
655835568	DALRD3	DALR anticodon-binding domain-containing protein 3	−0.87	1.22	1.40	−0.69	1.37	3.34
291396905	SMPDL3A	acid sphingomyelinase-like phosphodiesterase 3a	−0.76	−0.43	−0.93	−0.66	1.01	1.26
655895242	ARHGEF10L	rho guanine nucleotide exchange factor 10-like protein	3.28	−0.91	1.14	−0.65	−0.82	1.36
655902632	TNNT1	troponin T, slow skeletal muscle	2.50	−0.73	−0.81	−0.64	−0.69	−0.89
655897791	SH3BP5L	SH3 domain-binding protein 5-like isoform X2	−0.70	−0.44	−0.63	−0.64	−0.97	1.22
655706784	SLA2	src-like-adapter 2	−0.47	−0.62	−0.47	−0.64	−0.32	1.29
655902451	LAMA5	LOW QUALITY PROTEIN: laminin subunit alpha-5	−0.81	1.12	−0.96	−0.63	−0.65	−0.27
291405504	TMEM199	transmembrane protein 199	1.09	1.53	−0.91	−0.63	−0.94	2.42
655603581	CRY2	cryptochrome-2	1.13	−0.36	1.01	−0.63	−0.83	1.31
655729665	KRT7	LOW QUALITY PROTEIN: keratin, type II cytoskeletal 7	−0.73	1.43	−0.35	−0.63	−0.74	−0.92
655841393	CUL9	LOW QUALITY PROTEIN: cullin-9	1.35	−0.79	−0.52	−0.63	1.53	−0.48
655894938	PPP6R2	serine/threonine-protein phosphatase 6 regulatory subunit 2	−0.42	−0.55	−0.71	−0.63	1.06	1.86
291387257	CSNK1G3	casein kinase I isoform gamma-3 isoform X8	−0.91	−0.87	−0.89	−0.62	−0.85	4.01
291397177	TFB1M	dimethyladenosine transferase 1, mitochondrial	2.06	1.53	2.55	−0.60	1.74	1.32
291403160	ZNF106, ZFP106	zinc finger protein 106	1.01	−0.52	1.07	−0.55	1.09	−0.45
655867504	MRPL43	LOW QUALITY PROTEIN: 39S ribosomal protein L43, mitochondrial	1.19	1.13	−0.96	−0.53	1.03	−0.32
655765030	MB	LOW QUALITY PROTEIN: myoglobin	−0.91	−0.82	−0.81	−0.49	1.11	−0.81
655601432	#N/A	FXYD domain-containing ion transport regulator 6 isoform X3	−0.56	−0.38	1.05	−0.46	−0.93	−0.44
291389209		keratin, type II cytoskeletal 5 isoform X1	−0.63	1.11	−0.45	−0.46	−0.73	−0.76
655795749	DHODH	dihydroorotate dehydrogenase (quinone), mitochondrial	−0.75	−0.90	1.20	−0.46	−0.85	−0.91
291410767		histone H2A type 1-H	1.02	1.13	−0.50	−0.46	−0.83	1.33
655837047	GPNMB	transmembrane glycoprotein NMB	−0.58	1.19	−0.39	−0.46	−0.76	−0.66
655886422	MYLPF	myosin regulatory light chain 2, skeletal muscle isoform type 2	1.48	−0.33	−0.46	−0.45	−0.66	−0.68
291387979	XKR4	XK-related protein 4	−0.57	−0.74	−0.87	−0.45	1.04	1.54
655771366	NCCRP1	F-box only protein 50 isoform X2	1.59	−0.22	−0.46	−0.44	−0.39	−0.67
655828240	OSGEPL1	probable tRNA N6-adenosine threonylcarbamoyltransferase, mitochondrial isoform X2	1.01	−0.73	−0.94	−0.44	−0.38	−0.63
655871287	KRT10	keratin, type I cytoskeletal 10 isoform X2	−0.60	1.25	−0.33	−0.43	−0.92	−0.71
126723437	ENO3, ENO1	*beta-enolase	−0.68	−0.66	1.07	−0.43	−0.45	1.68
655835901	#N/A	band 4.1-like protein 3 isoform X19	−0.77	1.21	1.46	−0.41	−0.95	1.45
655831942	LATS2	serine/threonine-protein kinase LATS2 isoform X2	1.11	−0.62	1.07	−0.41	−0.81	1.07
655842035	OGFRL1	LOW QUALITY PROTEIN: opioid growth factor receptor-like protein 1	−0.29	1.02	−0.81	−0.38	−0.77	−0.50
655840529	C12H6orf136	uncharacterized protein C6orf136 homolog isoform X2	1.06	−0.52	−0.81	−0.38	1.09	1.44
291389971		keratin, type I cytoskeletal 18	−0.60	1.23	−0.28	−0.37	−0.64	−0.72
291387122	SMC6	structural maintenance of chromosomes protein 6	1.03	−0.60	−0.31	−0.36	−0.31	−0.52
291404357		hsc70-interacting protein	−0.45	−0.12	1.20	−0.34	1.08	−0.10
655839030	EMB	embigin	1.13	−0.36	1.19	−0.30	−0.76	−0.54
655877350	RAB9B	ras-related protein Rab-9B	1.23	1.79	1.67	−0.28	1.99	−0.41
655901361	ISG15	ubiquitin-like protein ISG15	−0.49	−0.94	−0.45	−0.28	−0.66	1.19
655897078	CD3EAP	DNA-directed RNA polymerase I subunit RPA34	1.90	−0.82	2.44	−0.25	1.99	1.54
655868318	NSMCE4A	LOW QUALITY PROTEIN: non-structural maintenance of chromosomes element 4 homolog A	1.76	1.38	1.86	2.44	1.99	−0.67

^*^Means the proteins are not predicted proteins.

^−^Means down-regulation.

While most of the protein presented a fold change less than 3 folds, VEGF receptor 1 (VEGFR1) was the only protein that showed more than a 10-fold increase. In the 0.5 mg-injected group, the protein level of VEGFR1 was 3.85 times, 3.32 times and 2.57 times higher than the untreated group at the 4^th^, 7^th^ and 14^th^ day respectively. In the 2.0 mg-injected group it was 18.35 times, 3.46 times and 6.73 times higher. The complement C3 alpha chain isoform X1 partial 1 and fibrinogen alpha chain were the second highest increased proteins. It reached a 5-fold increase at day 7 in the 0.2 mg injected rabbits. The most decreased protein was cyclic GMP-AMP synthase, which decreased to 0.11 fold at day 14 in the 0.5 mg group.

We especially examined the changes of growth factors and proteins associated with inflammation and apoptosis. Neither of those proteins presented a fold change greater than 2. VEGF-B, PlGF and interleukins including interleukin-1 and interleukin-17 were not identified.

**Figure 7:**
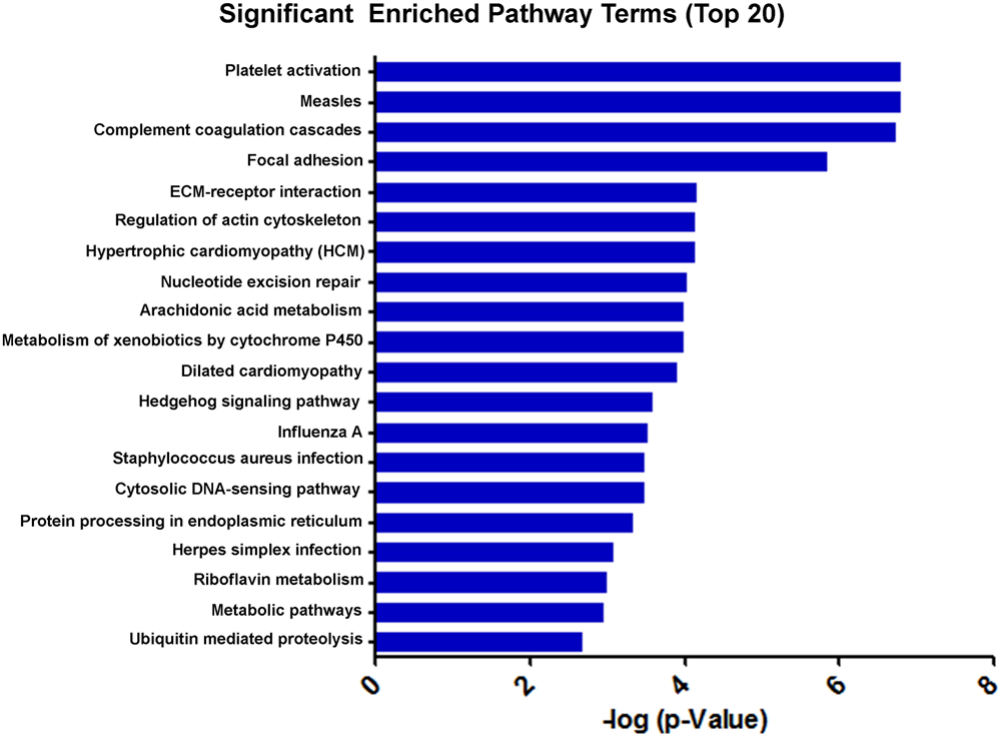
Differentially expressed proteins in the 6 study groups were enriched within 47 KEGG pathways compared to the untreated control group. The top 20 were presented.

To better understand the functions of the differentially expressed proteins, we performed KEGG pathway analysis. The top 20 significant enriched pathway terms are shown in Fig. 7.

## Discussion

We investigated the safety of intraocular injection of high concentration and high frequency of a novel anti-VEGF agent conbercept in rabbit. No drug-related side effects were detected with IOP, fundus, retinal morphology and function assessments. No apoptotic cells were found in the retinal GCL in the conbercept injected eyes. We did not observe alternations of the major pro-inflammatory cytokines. Considering the high concentration and high frequency intravitreal injection did not cause unwanted side effects, we concluded that conbercept is well tolerated in rabbit in a short term observation.

Most of the transient elevation of IOP after intravitreal injection is believed to be caused by a sudden increase of vitreous volume and can recover within a few minutes to a few hours^19^. However, it was reported that a limited number of AMD patients developed sustained elevation of IOP after single or repeated intravitreal injection of anti-VEGF drugs, which lasted from several weeks to even 1 or 2 years^20^. The increase of IOP at long-term might be attributed to the drug itself. Nevertheless, we did not observed elevation of IOP among all the groups in our study.

Although overproduction of VEGF is deleterious, adequate concentrations of VEGF may be important for the eye to maintain normal functions including vascular development and neurons survival^21–24^. PlGF, a member of the VEGF family, also exerts a role in the protection of neuron in the retina^16^. It was showed that potent inhibitors of all VEGF-A isoforms significantly diminished the protective effects of ischemic preconditioning on neurons. In addition, VEGF-A_120_ plays a supporting role in the survival of normal RGCs^22^. It was also reported that multiple injections of high doses of up to 5.0 mg bevacizumab in rabbits would induce photoreceptors apoptosis at 1 week after injections^25^. In a murine model, systemic administration of a viral vector expressing soluble VEGF receptor-1 led to a significant decline in ERG responses^26^. Since conbercept is a multi-target VEGF blocker and binds all isoforms of VEGF-A, VEGF-B, and PlGF with high affinity, it arouses a concern whether it will lead to unwanted retinal neuron death and dysfunction. The number of GCL cells and the thickness of INL were not changed and no TUNEL-positive cells were seen in the RGC layer of the conbercept-injected groups. Functionally, the ERG waveforms were normal and the amplitudes of the a- and b-waves were similar to the PBS injected control group. We did not observed retinal and choroidal structural abnormalities under light microscope. Thus, our data are in agreement with other studies^27^ that inhibition of VEGF or prolonged blockade of ocular VEGF receptors with conbercept would not cause morphological and functional damage to the retina.

Despite rarely happens, intraocular inflammation is a serious concern after intravitreal anti-VEGF treatment. We detected the protein expressions of IL-6, IL-8 and TNF-α in the aqueous humor of conbercept injected eyes. The levels of these inflammatory cytokines are similar to those in the PBS injected groups. Even though it is reasonable that the human fusion protein might cause inflammatory responses in rabbits, however, we did not observe such phenomenon.

We used 10-plex TMT-labeled proteomic quantification to analysis the variation of proteins in the retina of conbercept injected eyes. This technique has been demonstrated to be a powerful method to reach very large coverage of the proteome and to discover differentially expressed proteins (DEPs). Although the proteomic was based on a database of rabbit protein, it could help us to understand the protein alternation after intraocular administration of conbercept. To the best of our knowledge, this is the first study to explore DEPs in the retina after intraocular administration of an anti-VEGF drug.

Among 6042 proteins quantified, we identified 250 proteins (~4%) altered by more than 2.0-fold or less than 0.5-fold with greater than 95.0% probability at least at one time point or dosage in the eyes applied with 0.5 or 2.0 mg conbercept at day 4, 7 or 14. The only protein that reached more than 10-fold increases was VEGFR1. It was not surprise since conbercept is a recombinant fusion protein contains several ligand binding domains including VEGFR 1. Increase of VEGFR1 should be a consequence of increase of exogenous conbercept. On the other hand, the data proved the efficacy of the assay.

Proteomic analysis did not show significant changes of cell death or inflammation associated proteins in the conbercept-injected eyes. No increase of cytokine, chemokine or neuroinflammation related proteins was observed. Compared with the untreated group, conbercept did not cause substantial changes of the expressions of growth factors. Although the complement C3 alpha chain isoform X1 partial 1 was detected, no any other related proteins were found. In the complement and coagulation cascades pathway, the levels of three fibrinogens were higher than the control but these proteins are also related with platelet activation. Thus, alternation of the complement C3 alpha chain isoform X1 partial 1 maybe associated with platelet activation, rather than inflammation. Platelet activation was detected as the most enriched pathway after conbercept injection. Fibrinogen alpha chain, fibrinogen beta chain and fibrinogen gamma chain isoform X2 increased at day 7 after injection. It has been shown that application of ranibizumab and bevacizumab may contribute to a risk of systemic thromboembolic events in elderly patients^28^. Up-regulation of three fibrinogens after conbercept injection might raise a concern whether the proteins could be a potential risk factor. Research revealing the fibrinogen concentration in circulation would be helpful.

Mitochondrial adenosine triphosphate synthase (ATPase inhibitor) was up-regulated at all three time points. Bevacizumab was reported to show mild mitochondrial toxicity at clinically doses^29^. Five mitochondrial proteins were altered significantly with four up-regulated involved in steroid biosynthesis, lipoic acid metabolism, pyrimidine metabolism, glycerophospholipid metabolism and one down-regulated in amino acid metabolism.

In the protein processing in endoplasmic reticulum, alpha-crystallin A chain was approximately 2-fold at day 4 and 1.5-fold increased at day 7 and 14 after injected with both dosages of conbercept. Alpha-crystalline is a member of heat shock protein family and acts as chaperones which is acknowledged to be a neuroprotective substance^30^.

The Graves disease carrier protein (GDC) was down-regulated after conbercept injection at all three time points and decreased more than 5 folds in the 2.0 mg group at day 7. GDC is recognized in patients with active Graves disease (GD).

In the riboflavin (Vitamin B_2_) pathway, the expression of riboflavin (RF) kinase in the conbercept-injected groups decreased at all three time points. ATP:riboflavin kinase catalyzes the synthesis of cofactor flavinmononucleotide (FMN) by transforming riboflavin and ATP into FMN and ADP. RF is of physiological and nutritional importance in the maintenance of health of the retina^31^.

There are inevitable limitations for this study. First, we injected recombinant human fusion protein into the rabbit eyes. The data was obtained form a rabbit database and the protein information could not be completely used to predict the outcome in human. Second, the cutoff we set for protein changes is 2 folds. Thus we can’t exclude the possibility that a protein presents a fold change less than the threshold will not exert functional changes. However, since such investigation can’t be duplicated in human subjects, the first retinal proteomic study in anti-VEGF injected eye still provide important information with regard to the molecular changes in the retina. Based on these data, it is practical to confirm whether the proteins are actually altered and to explore their significances. In addition, it is also possible to decide whether supplement treatments are necessary. For example, if it is confirmed that riboflavin kinase activity is lower in anti-VEGF injected eyes and consequently causes unwanted effect, it might be necessary to supply the patients with flavinmononucleotide.

We concluded that intravitreal injection of high concentration and high frequency of conbercept is well tolerated at least in a short-term in rabbit. Our study offers a comprehensive and intuitionistic overlook on the alteration of protein expression in the retina injected with conbercept. The data provided important information for the future clinical study and for designing therapeutic protocols.

## Methods

### Animals

All experiments were conducted in accordance with the ARVO Statement for the Use of Animals in Ophthalmic and Vision Research and were approved by the Ethics Committee of The First Affiliated Hospital of Chongqing Medical University, Chongqing, China. The animals were fed with standard laboratory food and water in an air-conditioned room with a 12-hour light-dark cycle.

One hundred and eleven pigmented *Chinchila* rabbits, weighing 2 to 3 kg, were used. Only the right eye of each animal was injected and the left eye was untreated. Rabbits were randomized into nine groups. Group A (n = 3) did not receive any injection and was labeled as the blank. Group B (n = 27) received intravitreal injection of 50 μL of PBS and was labeled as control group. Groups C (n = 18), and D, E (n = 27 each) received intravitreal doses of 0.2, 0.5 and 2.0 mg/eye of conbercept on Day 0 respectively. Groups F and G (n = 6 each) received six weekly injections of 0.2 mg/eye of conbercept respectively. Groups H and I (n = 3 each) received 400 nmoles N-Methyl- D-Asparate (NMDA) and 100 μg lipopolysaccharide (LPS) respectively and were labeled as the positive control groups for TUNEL and ELISA essays.

The vitreous volume of a rabbit is approximately 1.5 mL and that of a human is about 5 mL. As the dose of 0.5 mg or less conbercept is frequently used in humans, the doses of 0.2, 0.5 and 2.0 mg for conbercept in rabbits are about 1.3, 3.3, and 13.3 times of that in human.

### Intravitreal injection

Intravitreal injection was performed in sterile conditions. Rabbits were anesthetized with injection of 3% phenobarbital sodium solution (30 mg/Kg) through the ear vein. After corneal surface anesthesia with oxybuprocaine hydrochloride eye drop (Santen Pharmaceutical Co., Ltd, Osaka, Japan), a 27-gauge needle attached to a 1 mL syringe was introduced into the vitreous cavity 3.5 mm posterior to the superotemporal limbus. The needle tip was directed towards the center of the vitreous under direct visualization. The conbercept solutions or PBS (50 μL) was slowly administered into the vitreous. To prevent reflux, the needle was held in place for 30 seconds before withdrawal. At the end of the procedure, lincomycin hydrochloride eye drops were applied.

### Intraocular pressure

At day 4, 7, 14 after the single intravitreal injection of conbercept (0.2 mg, 0.5 mg and 2.0 mg) or PBS, and at day 7 after the 6^th^ weekly-injection of conbercept (0.2 mg) or PBS, IOP was measured using a Schiotz tonometer (66 Vision Tech., Suzhou, China). Rabbits were anaesthetized with intraperitoneal injection of 1 mL/kg pentobarbital sodium. An average of five consecutive readings by the same observer was applied for analysis.

### Fundus Photography

The pupils were dilated with tropicamide eye drops (Shenyang Xingqi Pharmaceutical Co. Ltd, Shenyang, China) 30 minutes prior to imaging. The fundus photography of the rabbit eye was performed using a digital fundus camera system under anesthesia.

### Electroretinogram

Electroretinogram (RetiMINER System, AiErXi Medical Equipment Co., Ltd., Chongqing, China) was recorded at day 4, 7, 14 after the single intravitreal injection and at day 7 after the 6^th^ weekly intravitreal injection. After dark adaptation, rabbits were anesthetized with pentobarbital sodium. Pupils were dilated and Burian-Allen corneal bipolar electrodes (Hansen Laboratory, Iowa City, Iowa) were applied as the corneal electrodes. The ground electrode was placed subcutaneously on the back. Dark- and light-adapted ERGs were recorded followed a previous procedure^32^.

### Histological Evaluation

Animals were sacrificed with an injection of overdose sodium pentobarbital under deep anesthesia. Eyeballs were enucleated and half of the eyecup was fixed with 4% paraformaldehyde for 24 hours at room temperature^33^. Tissues were embedded in paraffin and 4-μm sections were cut through the optic disc and stained with hematoxylin and eosin (HE). The images of each section were acquired. The number of cells in the GCL was counted in a region of 800 to 1500 μm from the center of the optic nerve head on both sides. The thickness of inner nuclear layer (INL) was measured in three areas at a distance of 500 to 1000 μm from the edge of optic disc. Four sections of each eye were measured, and data were averaged for each eye. All measurements and analysis were performed in a masked manner.

### TUNEL

The terminal dUTP-mediated nick-end labeling (TUNEL) was performed to detect the apoptosis cells in the retina^33^. Sections were mounted with fluorescein-FRAGEL media. Staining was performed according to the manufacturer’s protocol (Roche Diagnostics, Mannheim, Germany). Samples were permeabilized in 100 μL of 20 μg/mL proteinase K for 20 minutes, equilibrated with 100 μL of 1% TDT buffer for 30 minutes at room temperature and labeled with 60 μL TDT labeling reaction mixture for 1 hour at 37 °C. Sections were photographed and the TUNEL-positive cells were counted between 1000 to 1500 μm from the center of the optic disc on both sides in the GCL and INL.

### ELISA

The concentrations of IL-6 (RayBiotech, Norcross, GA), IL-8 (R&D Systems, Minneapolis, Minnesota, CA) and TNF-α (RayBiotech) in 100 μL aqueous humor were determined according to the manufacturers’ protocols^33^. The absorbance at 450 nm wavelength was measured using a multifunction microplate reader (Molecular Devices).

### Protein Extraction

Retinas were isolated from the eyeball and frozen in liquid nitrogen and lysed using protein extraction buffer (8 M urea, 0.1% SDS) containing protease inhibitor cocktail (Roche, Indianapolis, IN, USA) on ice for 30 min and then centrifuged at 16,000 × g for 15 minutes at 4 °C. The supernatant was collected and protein concentration was determined by BCA assay kits (Pierce, Rockford, IL, USA).

### TMT labeling and fractionation of labeled peptide

Tandem mass tag (TMT) labeling was performed according to the manufacturer’s instructions (Pierce). Proteins were precipitated by pre-chilled (−20 °C) acetone. After resuspension, proteins were digested overnight at 37 °C by using 2.5 μg of trypsin. One tube of TMT10 Label Reagent was added to each 100 μg sample and the reaction was carried out at room temperature for 1 hour. After labeling, ten tissue samples were combined for one measurement. For fractionation of the labeled peptides, samples were first lyophilized and reconstituted. A total of 40 fractions were collected which were concatenated to 20 fractions, vacuum dried and stored at −80 °C until further analysis.

### LC-MS/MS Analysis

The LC-MS/MS analysis was carried out by Capitalbio Technology with a Q Exactive Mass Spectrometer (Thermo Scientific, San Jose, CA). Mass spectrometry analysis was performed in a data dependent manner with full scans (300–1,800 m/z) acquired using an Orbitrap mass analyzer at a mass resolution of 70,000 at 400 m/z in Q Exactive. Twenty most intense precursor ions from a survey scan were selected for MS/MS from each duty cycle and detected at a mass resolution of 35,000 at m/z of 400 in Orbitrap analyzer. All the tandem mass spectra were produced by higher-energy collision dissociation (HCD) method. Dynamic exclusion was set for 20 seconds.

### Data analysis

Proteome Discoverer software (Ver. 1.4, Thermo Scientific) was used to perform database searching against the *Oryctolagus cuniculus* database (46551 proteins) using the Sequest algorithms. Following settings were applied: precursor mass tolerance of 15 ppm, fragment mass tolerance of 20 mmu. Only high confident peptides with a global FDR < 1% based on a target-decoy approach were included in the results. In the TMT quantitation workflow the most confident centroid method was used with an integration window of 20 ppm. For protein quantitation, only unique peptides were used to quantify proteins.

Enriched pathways were analyzed in a command-line program KOBAS 2.0. We used the whole genome as the default background distribution to identify the significantly enriched pathways statistically in a set of sequences. For each pathway that occurs in the set of genes, we counted the total number of genes in the set that were involved in the pathway. We then calculated the *p* value using a hypergeometric distribution. If a whole genome has *N* total genes, among which *M* are involved in the pathway under investigation, and the set of genes has *n* total genes, among which *m* are involved in the same pathway, the *p* value for the pathway is calculated as follows:

Because a large number of KEGG pathways are considered, multiple hypotheses tests are performed. To reduce the Type-1 errors (i.e. false positive discoveries), we performed an FDR correction with a default cutoff of 0.05^34^.

### Statistical Analysis

The IOP results were presented as mean ± SD and the other data were mean ± SEM. Statistical analysis was performed using the GraphPad Prism (GraphPad Prism 5, Inc., San Diego, CA, USA). The results were analyzed by one-way ANOVA followed by Bonferroni correction for multiple comparisons. *p* less than 0.05 was considered statistically significant.
